# Pursuing Band-like Transport in Colloidal Quantum Dot Assemblies

**DOI:** 10.1021/acs.jpclett.5c04005

**Published:** 2026-04-08

**Authors:** Ricky Dwi Septianto, Dadan Suhendar, Mohammad Hamzah Fauzi, Satria Zulkarnaen Bisri

**Affiliations:** † Research Center for Quantum Physics, National Research and Innovation Agency (BRIN), South Tangerang 15314, Indonesia; § Department of Applied Physics and Chemical Engineering, 13125 Tokyo University of Agriculture and Technology, 2-24-16 Naka-cho, Koganei-shi, Tokyo 184-8588 Japan

## Abstract

Charge-carrier transport in semiconductor colloidal quantum dot (CQD) solids has been the subject of extensive debate and investigation for more than a decade. Understanding the underlying transport mechanisms in CQD assemblies is critical for unlocking their full potential in optoelectronic applications. To date, a widely accepted view holds that carrier transport in conventional ligand-capped glassy CQD solids is a non-adiabatic hopping process, supported by both experimental observations and theoretical models. In contrast, recent advances have enabled the self-assembly of CQDs into long-range-ordered superlattices with epitaxial connections between neighboring QDs. These superlattices are expected by many to exhibit band-like transport, potentially overcoming the limitations of hopping conduction in disordered systems. However, definitive experimental evidence and a comprehensive theoretical framework for charge transport in such highly ordered structures remain lacking. While the formation mechanisms of these superlattices have been widely explored, this Perspective aims to provide insight into the current understanding of charge-carrier transport in epitaxially connected CQD superlattices.

## Graphical abstract

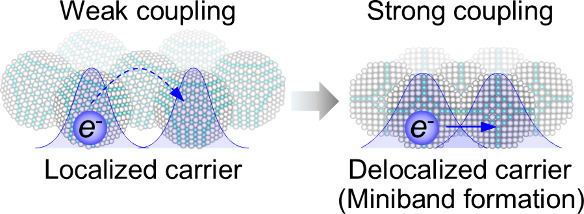

## Introduction

Semiconductor colloidal quantum dots (CQDs) are solution-processable nanoscale single crystals and are known for their tunable optical and electronic properties due to the strong confinement of the electron wavefunction. The size-dependent bandgap makes CQDs especially attractive for the next generation of optoelectronic applications,^1,2^ including light-emitting/display technologies,^3^ photovoltaics,^4^ and photodetectors.^5^ Since their initial discovery,^6,7^ the synthesis of CQDs has advanced significantly, enabling precise control over their size, shape, and composition, thereby allowing fine-tuning of the bandgap for light absorption and emission in the visible to mid-infrared range. Despite their remarkable promises, turning CQDs into high-performance thin-film electronic devices faces persistent fundamental challenges, particularly in understanding and improving charge carrier transport over CQD films.

For electronic applications, CQDs are typically processed into solid films by using solution-based deposition techniques. However, the formation of a solid film does not inherently result in electrical conductivity, as charge-carrier transport in CQD solids is primarily governed by electronic coupling between neighboring QDs. Long-chain organic ligands commonly contain the as-synthesized CQDs. These ligands, while essential during synthesis, act as insulating barriers that significantly weaken the electronic coupling between adjacent QDs, thereby limiting charge-carrier transport across the QD film. For years, tremendous efforts have been made to enhance the electrical conductance of CQD thin films by engineering their surface state, particularly by replacing long-chain ligands with shorter molecules to facilitate stronger interdot coupling.^8,9^ Under this condition, the charge carriers (electrons and holes) can travel from one QD to another QD site through hopping conduction.^10–13^

In recent years, electrical transport in CQD solids has been significantly improved. So far, carrier mobility has often been used as the standard metric to quantify this improvement. These values have been enhanced from typically on the order of 10^–5^ cm^2^ V^–1^ s^–1^ to achieve reportedly on the order of hundreds of cm^2^ V^–1^ s^–1^ (Table 1).^14–19^ Recently, electron mobility values of up to 270 cm^2^ V^–1^ s^–1^ from long-range DC conductivity measurements have been reported in a PbSe colloidal QD network,^20^ corresponding to approximately ∼27% of the bulk mobility (∼1000 cm^2^ V^–1^ cm^–1^).^21^ Notably, this value is remarkably close to the short-range electron mobility measured by terahertz (THz) conductivity performed on the same material.^22^ Hence, this substantial enhancement raises an important question: whether the carrier transport mechanism has transitioned from the hopping regime into band-like transport. Under strong electronic coupling, the significant overlap of extended carrier wavefunctions is expected to give rise to miniband formation, enabling band-like transport.^23–26^ Such a transport regime is believed to be crucial for boosting the performance of optoelectronic devices based on CQD solids. Nevertheless, the possibility of achieving band conduction in QD solids remains a topic of debate.^10,13,27^

**Table 1:** Reported High Mobility Values (>1 cm^2^ V^–1^ s^–1^) in CQD Solids

material	surface treatment/assembly type	measurement technique	carrier type^a^	mobility (cm^2^ V^–1^ s^–1^)	ref
PbS	epitaxial connection	FET,^c^ ion gel	e^–^	220	^19^
PbS	epitaxial connection, single monolayer	FET, ionic liquid	e^–^	10	^17^
PbSe	epitaxial connection	terahertz (THz)-frequency conductivity^b^	e^–^	260	^22^
PbSe	epitaxial connection	FET, ion gel	e^–^	278	^20^
PbSe	epitaxial connection	FET, Al_2_O_3_ gate dielectric	h^+^	6.5	^16^
PbSe	epitaxial connection	FET, ion gel	e^–^	24	^32^
PbSe	epitaxial connection with Na_2_Se–PbCl_2_ sequence	FET, Al_2_O_3_/SiO_2_ gate dielectric	e^–^	4.7	^41^
PbTe	NH_4_SCN	FET, SiO_2_ gate dielectric	e^–^	2.8	^9^
HgSe	As_2_S_3_	FET, SiO_2_ gate dielectric	e^–^	90	^53^
HgTe	hybrid ligand	FET, SiO_2_ gate dielectric	e^–^	65	^18^
HgTe	HgCl_2_	FET, SiO_2_ gate dielectric	e^–^	18.4	^15^
HgTe	HgCl_2_	FET, SiO_2_ gate dielectric	e^–^	8	^88^
HgTe	hybrid ligand	FET, SiO_2_ gate dielectric	e^–^	2.8	^120^
CdSe	epitaxial connection, ALD Al_2_O_3_ encapsulation	FET, SiO_2_ gate dielectric	e^–^	35	^14^
CdSe	NH_4_SCN	FET, Al_2_O_3_/SiO_2_ gate dielectric	e^–^	27	^42^
CdSe	NH_4_SCN	FET, SiO_2_ gate dielectric	e^–^	1.5	^9^

a e^–^: electron (n-type), h^+^: hole (p-type).

b FETs demonstrate long-range DC conductivity measurement.

c THz-frequency conductivity depicts short-range and ultrafast carrier (electron) transfer.

The pursuit of band-like transport in QD building blocks continues to attract significant attention. This interest has been motivated by the discovery of self-assembled QDs forming a superstructure with long-range order. In this system, the QDs, perceived as “artificial atoms”,^28^ are arranged in a manner similar to the actual atomic ordering in crystals. Furthermore, considering the different bonding strengths of ligands and the surface stoichiometry of QDs, it is now possible to form atomically coherent superlattices. These atomically coherent superlattices are found when the QD assemblies are “epitaxially connected” or “atomically fused” at specific crystalline facets.^29–31^ The emergence of such an epitaxially connected QD superlattice invites a re-examination of the underlying physical picture, whether the system still inherits the quantum confinement of individual QDs, as suggested by the phrase “connected but confined”,^29^ or possibly represents a fundamentally new transport phenomenon.^24^

This Perspective aims to provide a comprehensive overview of the current understanding of charge-carrier transport in QD solids by comparing two distinct systems. First, we revisit the established model for hopping transport in ligand-capped colloidal QD solids. This transport regime typically occurs in conventional QD solids, where the system exhibits insulating behavior due to relatively weak interdot coupling and strong electron localization. In other cases, the electron wavefunction can be extended across multiple QDs as their electronic coupling increases, creating a locally delocalized domain of charge carriers. Meanwhile, charge transport occurs between these domains via a hopping mechanism. The discussion then continues to the open-to-interpretation “touching” epitaxially connected QD superlattice framework. The hints for carrier transport in epitaxially connected QD superlattice systems will be elaborated, along with the criterion for achieving band-like transport. In this regime, the electrons are delocalized across the entire QD array, enabling charge carriers to propagate in a metal-like fashion. Finally, the future outlook on the importance and possible implications of achieving band-like transport will be discussed.

## Formation of CQD Solids

Over the past decade, considerable efforts have been made to enhance the electrical conductivity of CQD solids. As previously discussed, the native long-chain organic ligands (e.g., oleic acid) act as tunneling barriers, causing the CQD to be electrically insulating (Figure 1a). To overcome this issue, various surface modifications have been developed, including ligand engineering, selective ligand removal, and the application of an additional epitaxial layer. At the very beginning, the long-chain ligands were replaced by shorter molecules (Figure 1b) to enhance the coupling between adjacent QDs while preventing QDs from agglomeration. Further development employed inorganic ligands, such as halide or pseudohalide anions, to strengthen the electronic coupling (Figure 1c). More recently, a selective ligand-stripping process enabled QDs to come into direct contact at specific facets. This method led to the formation of long-range-ordered assemblies with high atomic coherence (Figure 1d). In such structures, strong interdot coupling is expected to give rise to miniband formation. However, whether quantum confinement is preserved in this regime remains a matter of debate, though some experimental evidence suggests that it is.^17,32^

**Figure 1:**
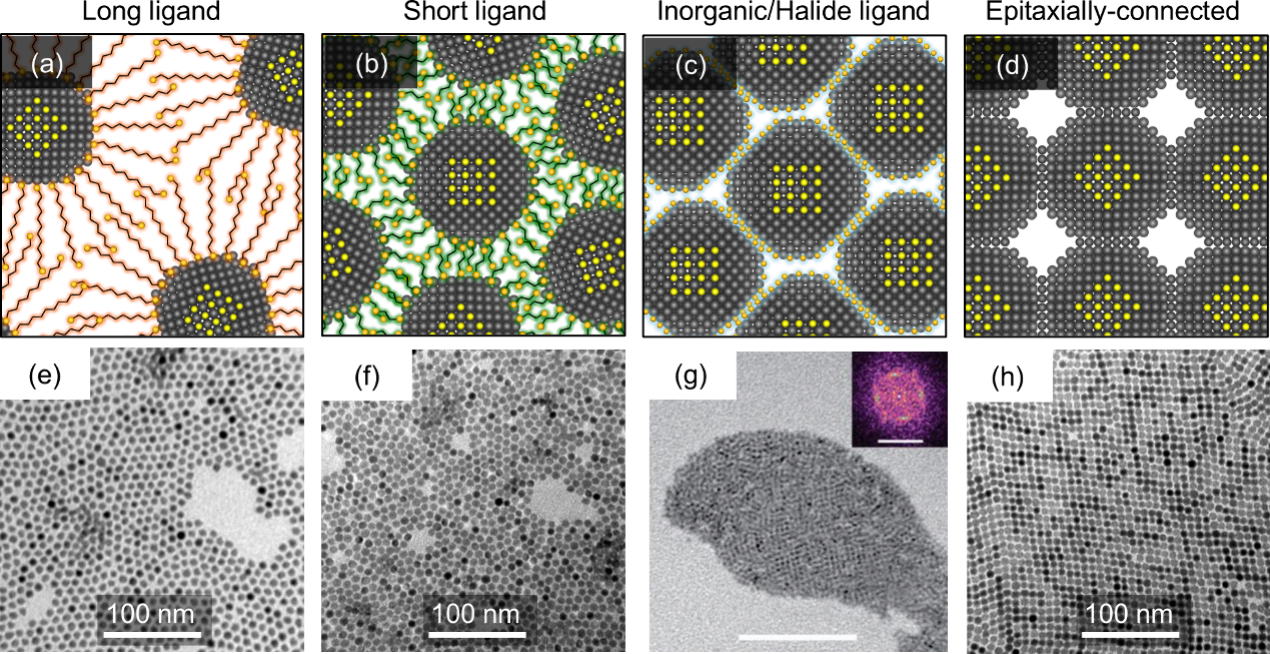
**Assembly of colloidal quantum dots.** (a–d) Illustrations of CQD assembly in different conditions: (a) capped with native long ligands; (b) capped with short molecular ligands; (c) capped with inorganic or halide ion ligands; (d) ligandless or epitaxially connected assemblies. (e–h) Corresponding electron microscope images of the PbS QD assemblies: (e) oleic acid-capped; (f) 1,2-ethanedithiol (EDT)-capped; (g) iodide-capped (from tetrabutylammonium iodide, TBAI); (h) epitaxially connected PbS CQDs. The scale bars are 100 nm. (e, f, h) Reproduced from ref 17. CC BY 4.0. (g) Reproduced from ref 43. Copyright 2018 American Chemical Society.

### Ligand-Capped CQD Solid

To make QD solids electrically conductive, shorter ligands replace the native long-chain ligand through a ligand-exchange process. So far, there are two standard techniques to proceed with the ligand exchange: (i) solid-phase ligand exchange^33,34^ and (ii) solution-phase ligand exchange.^35,36^ Various types of ligands have been investigated, such as short molecular ligands with different functional group terminations (−thiol, −carboxyl, etc.),^37–39^ inorganic ligands,^35,36,40,41^ pseudohalide (e.g., −SCN),^9,42^ and halide anions (e.g., I^–^, Br^–^, Cl^–^).^35,43^ Nevertheless, it was found that the capping ligand can significantly influence the energy landscape of CQDs; in other words, surface doping shifts energy levels.^44^

In solid-phase ligand exchange, the CQDs are deposited onto the substrate. Various solution-based deposition techniques have been employed to produce these CQD films, including spin-coating,^33,34^ dip-coating,^37,45^ and doctor-blading.^46^ In the best situation, a close-packed CQD thin film with the native ligand is then immersed in a solution containing the substitute short ligand with a sufficiently high concentration to promote ligand diffusion on the CQD surface that would allow surface reactivity competition with the native ligand. In earlier studies, short molecules such as hydrazine (N_2_H_4_),^8^ bidentate thiol-group molecules (e.g., 1,2-ethanedithiol (EDT) and 1,4-benzenedithiol (BDT)),^38,47^ bidentate dicarboxylic acid molecules,^39^ or alternating end-group molecules (e.g., 3-mercaptopropionic acid (MPA) and thiocyanate) were commonly used.^9,34,48^ Through these ligand exchange processes, the electronic coupling between neighboring QDs gets enhanced, resulting in improved electron and hole mobility values from typically less than 10^–5^ to ∼10^–1^ cm^2^ V^–1^ s^–1^,^1,49^ which are still too low for many expected applications. One of the culprits is the formation of macroscopic cracks in the films due to volume shrinkage during replacement of long ligands, thereby impeding charge-carrier transport. Many efforts were made to address this issue by refining the deposition method to allow subsequent filling of the formed cracks through a layer-by-layer CQD deposition process, one or a small number of monolayers at a time (Figure 2a).

**Figure 2:**
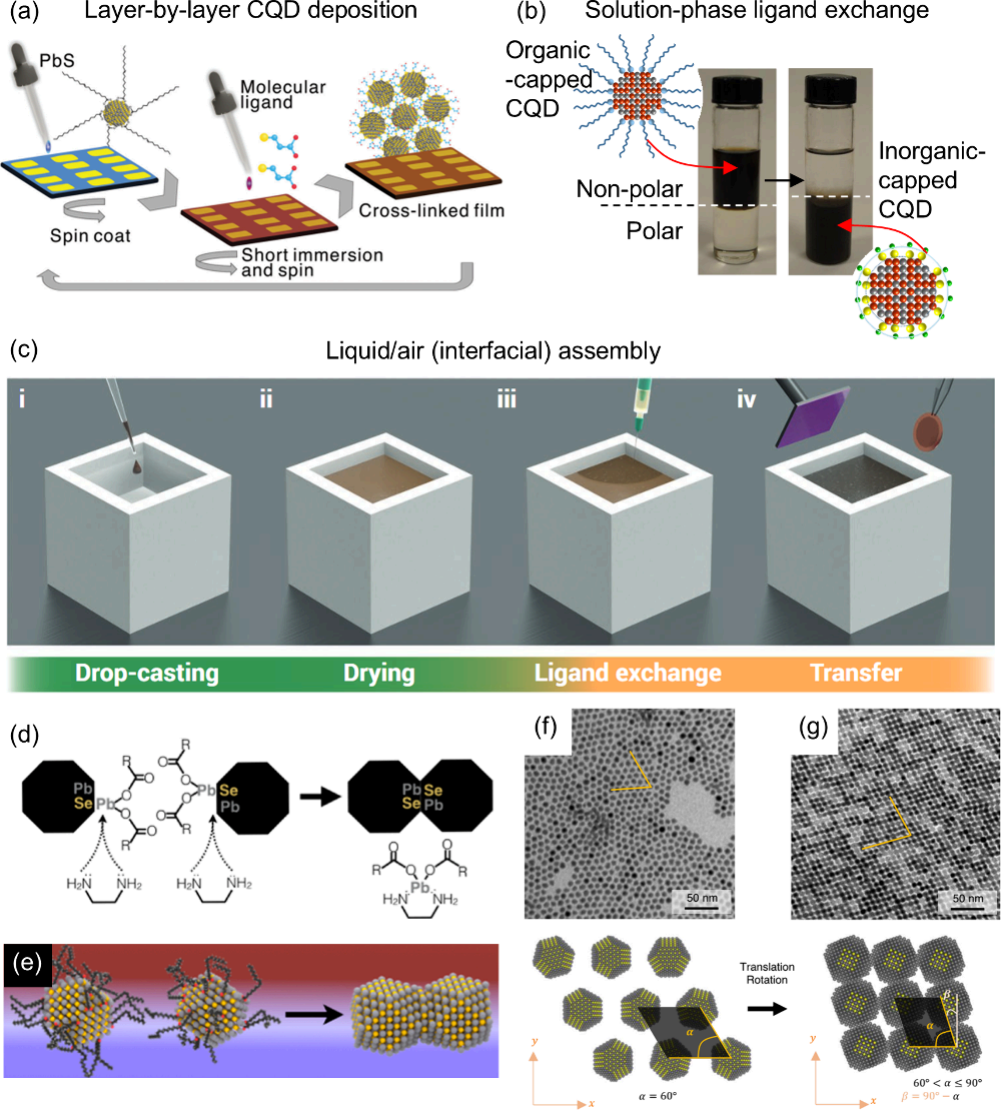
**Colloidal quantum dot solids.** (a) Illustrations of layer-by-layer CQD deposition via spin-coating method, including ligand-exchange process, (b) the solution-phase ligand exchange process, involving two separated-phase solution, and (c) schematics of liquid–air interfacial assembly to fabricate CQD superlattices based on the self-assembly process. (d, e) Selective ligand removal from the specific surface of PbS CQDs to form a square lattice. (f, g) Electron microscope images of PbS QD before and after selective ligand removal, showing hexagonal and rhombohedral lattices, respectively. (a) Reproduced with permission from ref 34. Copyright 2013 Wiley-VCH. (b) Adapted from ref 50. Copyright 2017 American Chemical Society. (c) Reproduced from ref 20. CC BY 4.0. (d, e) Adapted from ref 30. Copyright 2017 American Chemical Society. (f, g) Reproduced from ref 17. CC BY 4.0.

Although layer-by-layer techniques can enhance carrier transport by minimizing cracks in the CQD film, this method is effective only for producing a few robust layers. Meanwhile, for specific applications, such as solar cells, thick absorber layers (>100 nm) are preferred. Hence, another approach was to perform ligand exchange in solution. This technique enables a more robust ligand-exchange process, but it also requires maintaining the stability of the CQD solution.^36,50^ Figure 2b shows the typical solution-phase ligand exchange methods, mainly utilizing inorganic or halide ions as the final capping ligands. The process employs two immiscible solvents: polar and nonpolar. As the native organic surface ligand is replaced, the QDs are transferred from the nonpolar solvent into the polar solvent, resulting in a CQD ink after separation.^51^ A significant improvement has been demonstrated in this system, where mobilities can be enhanced (to become >1 cm^2^ V^–1^ s^–1^).^52,53^ In addition, a large area of film can be produced with this QD ink, suitable for upscaling device fabrication.

### Epitaxially Connected “Touching” QD Superlattice

CQD thin films fabricated by a conventional layer-by-layer technique often form a glassy or amorphous-like assembly due to rapid solvent evaporation.^54,55^ These conditions were identified as bottlenecks that limit efficient charge transport. Therefore, research efforts have been made to improve the quality of QD assembly and minimize spatial disorder in QD thin films, driven by QDs’ ability to self-assemble into superstructures.^56,57^ The individual QDs can self-assemble to make a long-range-ordered array, mimicking the atomic lattice in a crystal structure.^58,59^ This phenomenon has attracted interest, particularly from a physics research viewpoint. QD assemblies forming a long-range-ordered lattice with a well-defined structure might demonstrate cleaner and emergent transport as collective behavior.

So far, the epitaxially connected superlattice assembly has been successfully demonstrated in rock-salt Pb–chalcogenide QDs, due to their well-defined surface atoms. The self-assembly mechanism has been extensively studied to understand the natural formation of these QD superstructures. By achieving surface-necked connections among individual QDs, well-ordered superlattices can be formed with high atomic lattice coherence, also known as atomically fused or epitaxially connected QD superlattices. While the solid protocol and several factors influencing assembly formation have been intensively discussed,^31,60–65^ the formation of an epitaxially connected QD superlattice can be divided into two main stages. The first stage consists of the self-assembly process of the CQD at the liquid–liquid interface. The deposition of CQDs on the liquid surface provides a high degree of freedom for QDs to move, resulting in more compact assemblies as the native solvent completely evaporates. The method is based on well-known Langmuir–Schaefer method,^59,66,61^ which later has been called the liquid–air interfacial method within the QD community (Figure 2c). For this purpose, two immiscible solvents are typically utilized, where the QD with a native nonpolar solvent such as hexane, heptane, etc., is dropped on the top of a polar solvent like dimethyl sulfoxide (DMSO) or ethylene glycol (EG) as a flexible substrate. This condition facilitates a high degree of freedom for QDs to self-assemble in the subphase. In the second stage, an L-type ligand such as ethylenediamine (EDA) is introduced and injected into the bottom solvent to initialize selective ligand removal from the specific surface, forming a preferred assembly structure (Figure 2d).

In rock-salt Pb–chalcogenide QDs, the particles tend to reshape into truncated cuboctahedra, primarily dominated by two of the crystal facets, {100} and {111}.^67^ The native oleate ligands bind to these facets with different binding strengths, attaching more weakly to the {100} surfaces than to the {111} surfaces (Figure 2e). Hence, this asymmetry enables the selective removal of ligands from the {100} facet, which in turn promotes direct connections between neighboring QDs through these surfaces. As a consequence, the “square” lattice arrangements can be formed. For specific sizes, epitaxially connected QD assemblies exhibit incomplete square lattices or transform into rhombohedral structures, where the angles between the lattice sides deviate from 90° (Figure 2f,g).^17^ This distortion likely arises from slight misalignments (or offsets in the surface atom positions) between adjacent {100} facets. Beyond single layers,^17,68^ studies have also shown that these QDs can stack epitaxially into multilayer superlattices, yielding three-dimensional architectures.^20,62,69,70^ Such thick epitaxially connected QD solids open new opportunities for applications that demand robust, densely packed QD solids.

## Transport in Ligand-Capped CQD Solids

In this section, we highlight the current understanding of the transport mechanism governing carrier transport within QD solids. In the ligand-passivated QD solid, the framework of the hopping mechanism has been theoretically deliberated and experimentally demonstrated.^10–13,71–73^ Two distinct hopping mechanisms in QD solids have been observed depending on the operating temperature: nearest-neighbor hopping (NNH) at high temperatures (Figure 3a),^11,71,72^ and variable-range hopping (VRH) in the lower-temperature regime (Figure 3b).^12^

**Figure 3:**
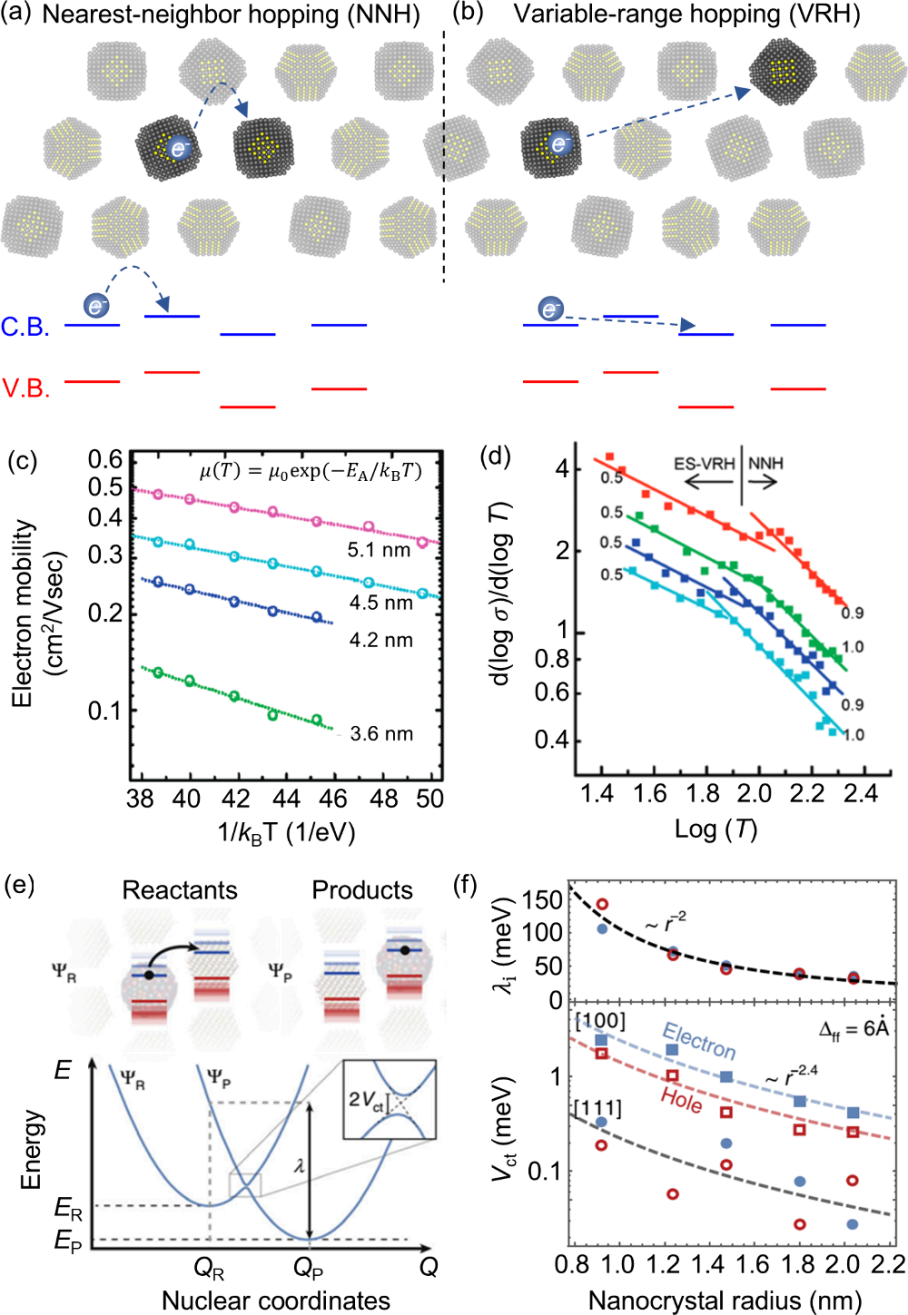
**Carrier transport in the weak coupling regime.** (a, b) Two possible hopping mechanisms in weak-coupling QD solids: (a) nearest-neighbor hopping (NNH) and (b) variable-range hopping (VRH). (c) Temperature-dependent mobility of PbSe CQD solids, showing Arrhenius behavior with activation energy *E*_a_ as a function of size. (d) Transition from NNH to VRH regime as a function of temperature. (e, f) The small polaron-assisted hopping mechanism at high temperature demonstrates the influence of electronic coupling and reorganization energy, which is size-dependent. (c) Adapted from ref 72. Copyright 2010 American Chemical Society. (d) Adapted from ref 11. Copyright 2011 American Chemical Society. (e, f) Adapted from ref 76. CC BY 4.0.

In general, the charge carrier transport in CQD solids is determined by the energetic landscape of the system, where disorder plays a crucial role. Several key factors contributing to the carrier transport are (i) fluctuation of the energy level of the site due to size distribution (variation in band gap) and number of donors (shifting the chemical potential), (ii) the charging energy, i.e., the energy to add (remove) a charge carrier to (from) a QD site, and (iii) the coupling energy, which determines the wavefunction overlap between neighboring QDs. In general, charge transfer in QD solids is an intricate competition between energetic disorder and coupling energy. Therefore, much of the macroscopic parameter (i.e., mobility) is sensitive to external influences (e.g., temperature and electric field).^40,74^ At the same time, these energetic disorder and coupling energy parameters are also associated with the size of the QD and the interparticle spacing.

## Transport in the High-Temperature Regime

In the high-temperature regime, including room-temperature level, charge carriers are provided with sufficient thermal energy to hop between neighboring QDs via the nearest-neighbor hopping mechanism, as illustrated in Figure 3a. The mobility of hopping conduction follows Arrhenius behavior, described by the relation μ = μ_0_ exp­(−*E*_a_/*k*_B_ *T*), where μ_0_ is the pre-exponential factor, *E*_a_ is the activation energy, *k*_B_ is the Boltzmann constant, and *T* is the temperature. The pre-exponential factor μ_0_ might include information on the interparticle spacing *l*, which is equal to the ligand length that separates adjacent QDs, according to the expression μ_0_ ∝ exp­(−β*l*), where β is the tunneling decay constant. This ligand length effect has been acknowledged in alkyl-chain-capped PbSe QD solids, where the mobility increased by 2 orders of magnitude as the interparticle spacing or alkane chain was reduced to half, indicating β to be ∼1.1 Å^–1^ for alkane-type ligands.^71^

On the other hand, the activation energy *E*_a_ was found to be size-dependent. Figure 3c shows the temperature-dependent mobility of various-sized CdSe QD solids, which follows Arrhenius behavior. The *E*_a_ value decreases from approximately 55 meV to around 30 meV as the QD size increases from 3.6 to 5.1 nm.^72^ Similar NNH behavior has also been observed in 1,2-ethanedithiol (EDT)-capped PbSe QD solids over a temperature range of 200 to 30 K, showing consistent size-dependent *E*_a_.^11^ Reducing *E*_a_ enhances the carrier mobility. However, it was found that the mobility does not increase monotonically with QD size but rather demonstrates a turning point at which mobility begins to decrease with increasing QD size.^71,75^ To explain this phenomenon, we must consider another important energy parameter, which is the coupling energy.^73,75,76^ Taking into account this parameter, the negative gradient of size-dependent mobility can be reproduced, as demonstrated by the relatively large size of PbS and PbSe QDs.^71,73^

### Polaron-Assisted Hopping Transport

In the most recent study, the carrier-transport mechanism has been comprehensively elucidated by accounting for the influence of polarons in QDs.^76^ The hopping rate τ_pol_^–1^ in the existence of small polarons follows Marcus hopping model:^76^

1 $${\tau_{\mathrm{pol}}}^{-1}=N_{P}\frac{2\pi }{\hbar }{V_{\mathrm{CT}}}^{2}\sqrt{\frac{1}{4\pi \lambda k_{B}T}}\;\exp\left[-\frac{{\left(\Delta E+\lambda \right)}^{2}}{4\lambda k_{B}T}\right]$$

where Δ*E* is the energy difference between the two sites, *N*_P_ is the degeneracy of the states activated by donor–acceptor interaction, providing the pathway for electron transfer,^77^ and *V*_CT_ and λ are the electronic coupling (coupling energy) and reorganization energy (Figure 3e), respectively. In QDs capped with an X-type ligand, which involves an acceptor–donor interaction,^44,78^ the ligand position can shift away from the surface as the ligand interacts with electrons in the QD, leading to the formation of polarons. During charge transfer from one QD to another, a rearrangement of the surface states occurs. The energy associated with this structural adjustment is λ. The reorganization energy has been found to be an order of magnitude larger than the electronic coupling (Figure 3f), implying that the charge carrier is localized in an individual QD due to the polaron.

Both the reorganization energy and coupling energy decrease as the QD size increases.^76^ While the influence of polarons becomes less significant in a large QD, the transfer rate or mobility of the charge carrier will be determined by the coupling energy *V*_CT_ (μ ∝ *V*_CT_^2^). This situation provides a reasonable explanation for the previously discussed negative correlation between QD size and carrier mobility. Moreover, it has also been demonstrated that electronic coupling is facet-dependent. At identical interparticle spacing, the electronic coupling through the {111} facet is approximately 1 order of magnitude lower than that through the {100} facet.^76^ This observation suggests anisotropic transport in which the spatial orientation of QDs within the array plays a significant role in determining the charge transport behavior.

### Transport at Low Temperature

At sufficiently low temperatures, charge-carrier transport in QD solids deviates from Arrhenius behavior, transitioning to the VRH mechanism (Figure 3b,d).^11,12,79^ In the VRH regime, charge carriers can “tunnel” across several localization states to find a favorable energy site. The probability of carrier tunneling from one localized site to another at a given distance is determined by the localization length ξ. Mott formulated the hopping conductance at very low temperatures, where the states contributing to carrier transport lie within a narrow band near the Fermi level. The derived temperature-dependent conductance follows Mott’s law:^80^

2 $$G\left(T\right)\propto \exp\left[-{\left(\frac{T_{0}}{T}\right)}^{1/4}\right]$$

where

3 $$T_{0}=\frac{\beta }{k_{B}g_{0}\xi^{3}}$$

in which *g*_0_ is the density of states at the Fermi level and β is a numerical coefficient. In Mott’s law, the density of states near the Fermi level is assumed to be constant. When the existence of a soft Coulomb gap due to electron–electron interactions is present, a correction to Mott’s law should be considered. This correction was proposed by Efros and Shklovskii, leading to the Efros–Shklovskii VRH (ES-VRH) mechanism, in which the temperature dependence is given by^81^

4 $$G\left(T\right)\propto \exp\left[-{\left(\frac{T_{\mathrm{ES}}}{T}\right)}^{1/2}\right]$$

where *T*_ES_ = β_1_ *e* ^2^/κξ, in which β_1_ and κ are the numerical coefficient and the dielectric constant, respectively.

In most cases, the carrier transport in CQD solids at low temperature follows the ES-VRH mechanism.^11,12^ However, the interesting Mott–ES VRH crossover has been observed in CdSe QD solids, which is found to be doping-level-dependent.^12^ VRH was observed at temperatures lower than 110 K. The doping level was controlled, ranging from less than 1 electron per dot (e^–^/dot) to approximately 6 e^–^/dot. The doping level was controlled and monitored through a spectro-electrochemistry measurement. The accumulated carrier density in QD solids was induced electrochemically, and the response to doping or chemical potential shifts was indicated by bleaching of the corresponding excitonic peaks. ES-VRH was reported to fully occur when the Fermi level is at half filling of the 1s_e_ band (∼1 e^–^/dot) or at the 1p_e_ state (doping level > 3 e^–^/dot). As the Fermi level lies below the 1s_e_ state (doping <1 e^–^/dot) or in the gap between the 1s_e_ and 1p_e_ states (fully filling the 1s_e_ state, 2–3 e^–^/dot), the transport exhibited Mott–ES crossover. The localization length ξ was estimated to be approximately 1.1 nm, which is much smaller than the QD size used in the experiment (∼6.5 nm).

ES-VRH was also observed in 1,2-ethanedithiol (EDT)-capped PbSe QD solids. The carrier transport exhibits ES-VRH behavior as the temperature is lowered below 100 K, transitioning from NNH in the higher-temperature regime (Figure 3d).^11^ In this PbSe, the localization length was reported to be comparable to the size of the QDs, and it scales with the size of the QDs. The carrier density was estimated to be around 4 × 10^12^ e^–^/cm^2^, corresponding to approximately 2 e^–^/dot at the QD with a size of 7.1 nm. According to the estimated doping level, the Fermi level should be within the 1s_e_ state of the conduction band in PbSe QDs, considering that the PbX system has 4-fold degeneracy, which results in 4 e^–^/dot for 1s_e_ half-filling and 8 e^–^/dot for complete filling. In addition, considering that the half filling and complete filling of states in QD assemblies is crucial, this factor will also be discussed later within different transport frameworks.

## Pursuing Band-like Transport in CQD Solids

In band-like transport, charge carriers (electrons or holes) are treated as mobile particles with extended wavefunctions throughout the material, behaving as Bloch states. This condition contrasts with the hopping regime, where the carrier wavefunction is localized within a single QD and decays exponentially beyond its boundary. When discussing band-like transport in QD solids, the central challenge is to shift from a conventional hopping-based perspective to one that accounts for charge-carrier delocalization. Therefore, a key objective is to provide evidence that electrons are no longer confined to individual QDs but are instead delocalized across multiple sites. It has been argued that to realize metallic conductance in QD solids, a large conductance channel and low energy disorder must be achieved in the first place. It will allow high electron transmittance to pass through the QD array. Hence, the criteria for achieving metallic conduction should be rigorously assessed.^10^

### Band Structure

The term quantum dot refers to a nanocrystal with strong confinement of the electron wavefunction within. However, as individual QDs come into proximity, in extreme conditions of surface contact (epitaxial connection), a miniband is formed due to the extended wavefunction of the electron. This raises a critical question: can such assemblies still be classified as quantum dots, or is an emerging low-dimensional system created from this epitaxial connection system instead? Kalesaki et al. presented an interesting perspective through a tight-binding calculation to determine the band structure of epitaxially connected QD superlattices.^24^ The epitaxially connected QD superlattices have a unique positioning between bulk and isolated QDs. From the bulk viewpoint, the truncation geometry in the epitaxially connected superlattices (Figure 1d,h) induces electronic wave scattering, leading to the opening of gaps. It is a sort of “confinement”. On the other hand, if we observe it from the isolated QD viewpoint, the band structure of the epitaxially connected QD superlattice is more dispersed. For example, in the conduction band, the higher energy level (1p_e_) has a broad bandwidth while still maintaining a gap to the lower energy level (1s_e_). If this model accurately represents the actual system, the resulting analysis might be quite interesting, as we discuss later from another perspective how these energy states can influence carrier transport. In addition, a similar tight-binding calculation was performed to demonstrate variation in the band structure of an epitaxially connected QD superlattice as it attached from different facets.^25^

### Localized versus Delocalized Charge Carriers

To further discuss the transport mechanism and the transition from hopping to band-like transport, we need to define a physical measure to distinguish the charge-carrier localization/delocalization regime. In QD assemblies, we start with a highly disordered system; consequently, the carriers are strongly localized within the individual QDs. On the other hand, the delocalization of charge carriers might be defined as the extension of the electron wavefunction across multiple QD arrays. It is characterized by the localization length ξ, which defines the exponential decay of the electron wavefunction in the disordered system. In general, ξ is influenced by the spatial and disorder parameters and the charge-carrier density.^82–84^ The higher the energetic disorder, the greater is the carrier density needed to achieve delocalization (extended ξ). The delocalization of charge carriers can occur when the localization length ξ is greater than the size of QD (ξ/*d* > 1).^84,85^ While this argument might be partially accepted, band-like transport can be achieved as delocalization of the electron wavefunction occurs within the entire crystal domain in disordered semiconductors.^86^ It is when the localization length diverges (ξ → ∞) and the conductance reaches quantum conductance limit (see discussion in Box [Boxed-text box1]).^87^ Therefore, a critical question remains: whether QD solids have the possibility to achieve this divergence of localization length, and if so, what criterion is needed to realize such a condition.

1 **Metal-to-Insulator Transition in Low-Dimensional Materials**

In low-dimensional materials or disordered systems, quantum interference can induce electron localization, known as Anderson localization.^81,149^ The key parameter governing charge transport in such a system is the localization length ξ, which characterizes the spatial extent of electronic wavefunctions. In the insulating regime, where ξ is smaller than the size of the system *L* (ξ < *L*), the electron transport or conductance decays exponentially, following *G* ∝ e^–*L*/ξ^. As the degree of disorder is reduced or the carrier density increases, the localization length grows and can become larger than the system size. In an ideal condition, ξ → ∞. This condition allows the electron wavefunction to effectively extend across the system, leading to a crossover from an insulating state to a metallic state (or band conduction in semiconductor materials). In the regime where ξ ≫ *L*, the transport is well-described by the Landauer formula, and the conductance approaches the quantum conductance limit *G*_m_ = *e* ^2^/πℏ, corresponding to a small number of phase-coherent conducting channels. Thus, a localization length comparable to the system size indicates the threshold between localized (insulator) and extended states.

This crossover is also reflected in low-temperature conductance behavior. In the band transport (metallic-like) regime, the conductance typically demonstrates a negative temperature coefficient (d*G*/d*T* < 0) and remains finite at *T* → 0 K. In contrast, in the localized states, the transport is dominated by a hopping mechanism, yielding d*G*/d*T* > 0 and *G* = 0 at *T* → 0 K. However, the true metallic state is subtle and is often influenced by additional effects, such as electron–electron interactions, particularly in 2D and quasi-2D systems.^87,119^

To help analyze this matter, let us first compare some experimental examples. Looking back at CdSe QD solids that showed VRH transport, the localization length was found to be 1.1 nm, which was smaller than the QD size (∼6.5 nm) with the additional 0.8 nm interparticle separation. This means that the electron is strongly localized and its wavefunction decays rapidly outside a single QD, putting this system in the weak-coupling regime.^12^ On the other hand, a quite large localization length (∼39 nm) has been observed in HgTe solids with ∼13 nm QD size (ξ ≈ 3*d*).^88^ In this case, the electron is “delocalized” within the local domain consisting of a few QDs, and a hopping mechanism emerges between neighboring domains (Figure 4b). In fact, this kind of behavior has been observed in highly doped organic semiconductors.^89^

**Figure 4:**
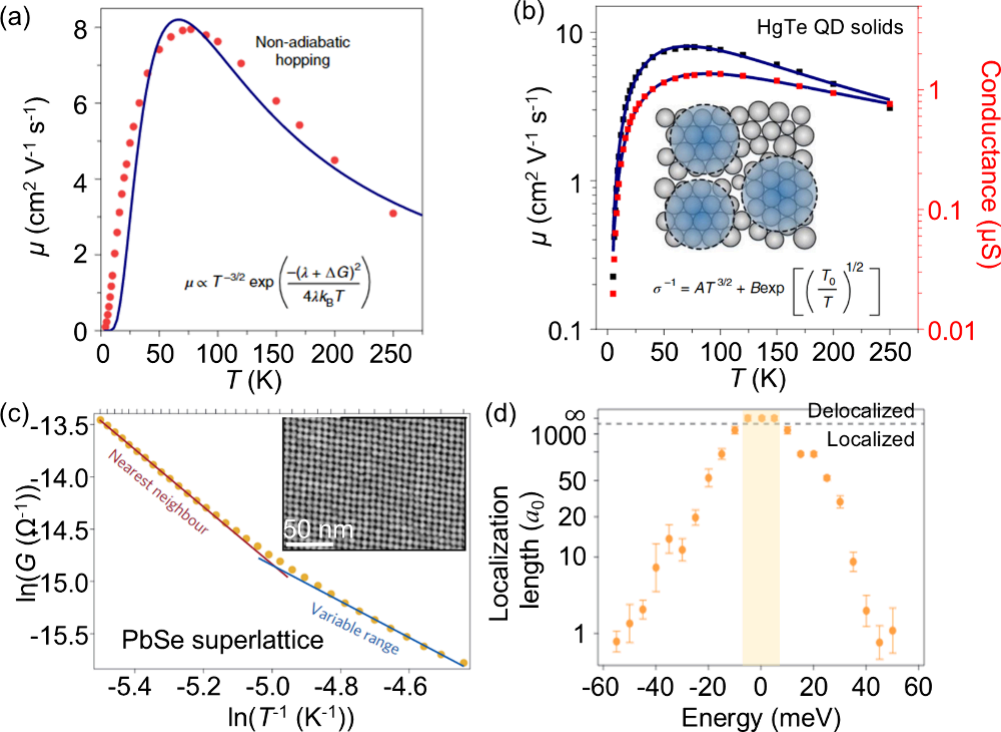
**Localization vs delocalization of charge carriers.** (a, b) Temperature-dependent mobility of HgTe CQD solids. The simple polaron-assisted fitting (non-adiabatic hopping) in (a) shows disagreement with the experimental data, whereas applying a local domain delocalization model results in the best fit (b). (c) Transport in epitaxially connected PbSe superlattices, which inherits the transition from NNH to VRH, whereas the theoretical prediction in (d) tells that the delocalization can occur at zero epitaxial connection disorder and 100% connectivity with permissible size dispersion. The zero of energy refers to the average value of the 1s_e_ energy level distribution due to size dispersity, and the *a*_0_ unit represents the normalized localization length (dimensionless) with respect to the QD radius. (a, b) Adapted with permission from ref 88. Copyright 2020 the authors of ref 88, under exclusive license to Springer Nature. (c, d) Adapted with permission from ref 90. Copyright 2016 Springer Nature.

In the last example, Whitham et al. investigated charge transport in three-dimensional, epitaxially connected PbSe superlattices using a SiO_2_-gated field-effect transistor (FET).^90^ Their study showed that the transport mechanism of the system aligned with NNH near room temperature and transitioned to VRH at lower temperatures (Figure 4c), with a calculated localization length of ∼10 nm from 6.5 nm size QD superlattices. Despite the expected strong electronic coupling facilitated by surface connections and highly coherent atomic lattices, the obtained localization length ξ/*d* was not significantly higher than that obtained in the glassy HgTe CQD solids.

Two possible reasons might explain this situation. The first reason is a different level of energetic disorder caused by size dispersion, electronic coupling disorder due to variations in connection width, and connectivity disorder. All these factors promote interference in the electron wavefunction, which can create localized states, exhibiting Anderson localization. Based on calculations, when coupling fluctuations and connectivity disorder are significantly suppressed, band conduction becomes achievable (Figure 4d), even in the presence of fluctuation energy arising from size dispersion.^90^ Given the progress in synthetic techniques, a size dispersity of 3–5% is currently achievable for some of the established CQD compounds.

The second reason is the charge carrier density. The number of doping levels influences the determination of the localization length and thus the transport mechanism. We will discuss how important this carrier density is in order to realize band-like transport in QD solids, especially epitaxially connected QD superlattices, in the following subsection.

### Metal-to-Insulator Criterion

To discuss the transition from insulator to metallic behavior with regard to achieving band-like transport, we can also consider it from the metallic side to derive the required parameter. In the metallic state, the localization length diverges. In this condition, the conductance of the QD array (*G*) approaches the quantum conductance limit (*G*_m_ = *e* ^2^/πℏ). Hence, Chen et al. quasi-classically derived a criterion for achieving the metal-to-insulator transition (MIT) in epitaxially connected QD superlattices.^85^ For a “touching” QD with connection width 2ρ (Figure 5a), the electrical conductance can be expressed as *G* = (*ek*_F_ρ)^2^/4πℏ, in which *k*_F_ = (3π^2^ *n*/*g*)^1/3^ is the Fermi wavevector of electron gas, *n* is the carrier density, and *g* is the degeneracy. For a spherical model, *n* = 6*N*/π*d* ^3^, where *N* is the number of electrons per dot and *d* is the QD size. Thus, at the quantum conductance limit, one can obtain the critical carrier density *n*_c_ that satisfies *n*_c_ρ^3^ ≈ 0.3*g*.

**Figure 5:**
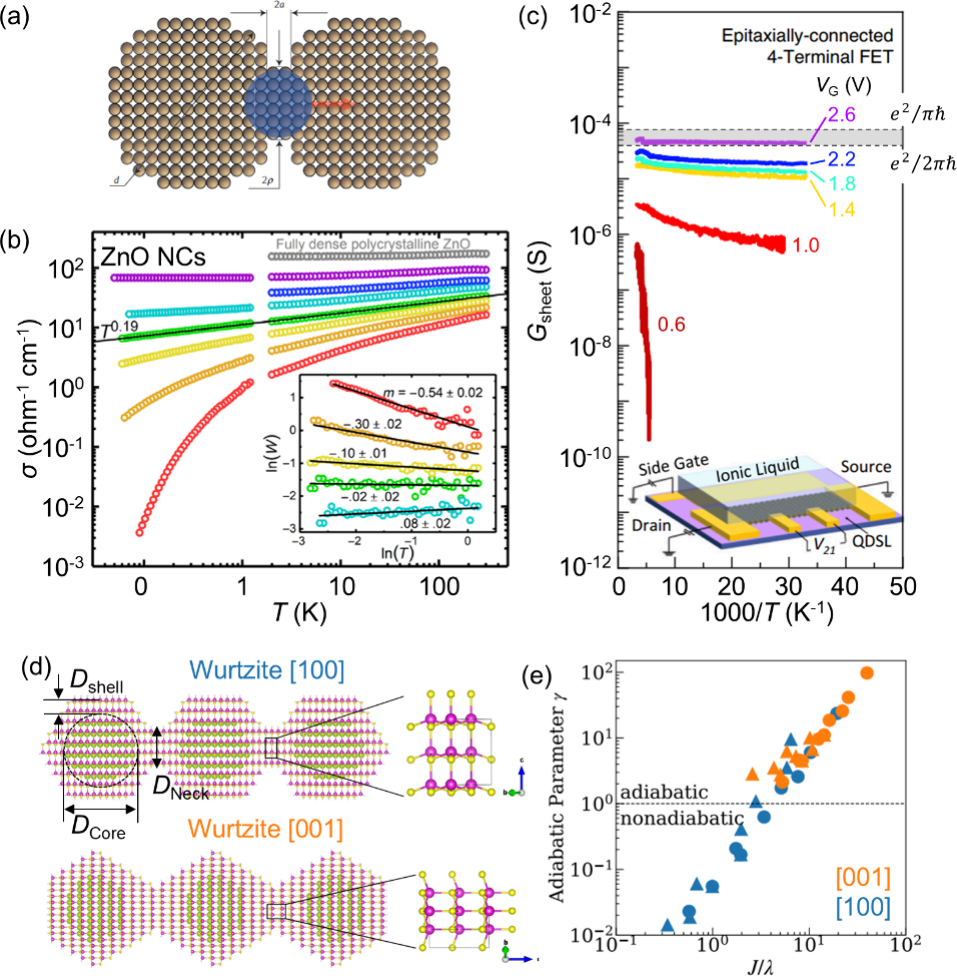
**Metallic-to-insulator transition (MIT) in CQD solids.** (a) Condition of the MIT criterion in epitaxially connected QD solids. The critical carrier density (*n*_c_) is determined by the connection width ρ. (b) MIT was observed in a photoinduced doping ZnO nanocrystal (NC) network. (c) Similar observation of MIT in an epitaxially connected PbS QD superlattice through electrostatic doping, generated by electric-double-layer (EDL) gating. The sheet conductance approaches the quantum conductance limit at *V*_G_ = 2.6 V. (d, e) Theoretical prediction of the transport transition from the non-adiabatic (hopping) to adiabatic (band-like/metallic) regime through the perspective of electron–phonon coupling. (a) Reproduced with permission from ref 85. Copyright 2015 Springer Nature. (b) Reproduced from ref 96. CC BY 4.0. (c) Adapted from ref 17. CC BY 4.0. (d) Adapted from ref 105. Copyright 2025 American Chemical Society. (e) Reproduced from ref 104. CC BY 4.0.

Based on this MIT criterion, for a system containing 6.5 nm diameter truncated cuboctahedral PbSe QDs, which have 4-fold state degeneracies (*g* = 4) with an average connection width (2ρ) of ∼3.26 nm, the critical carrier density *n*_c_ is estimated to be ∼2.8 × 10^20^ cm^–3^. This value corresponds to *N* ∼ 40 e^–^/dot, implying complete occupancy of the 1s_e_ state (8 electrons) and 1p_e_ state (24 electrons), with the remaining 8 electrons fill the 1d_e_ state. Meanwhile, assuming effective charge accumulation at the aforementioned experimental result of the PbSe QD superlattice with a SiO_2_ gate (200 nm SiO_2_, *C*_ins_ ≈ 17.25 nF cm^–2^) and two-dimensional QD density of approximately 2.3 × 10^12^ dots cm^–2^, the estimated maximum doping level is ∼2 e^–^/dot at an applied gate voltage of 40 V.^90^ The number of electrons per QD was 1 order of magnitude below the threshold for achieving the MIT condition.

On the other hand, for nondegenerate systems such as Cd– and Hg–chalcogenide QDs^91–93^ with the same QD size and contact radius, *n*_c_ is estimated to be ∼10 electrons per dot, corresponding to full occupancy of the 1s_e_ state and the 1p_e_ state. These estimations suggest that the MIT could occur when the Fermi level approaches higher energy levels. It is interesting in this regard that band transport can be achieved when the number of electrons is sufficient to fully occupy the 1p_e_ state, which coincidentally matches the broader bandwidth (more conducting channel) of the 1p_e_ state as predicted by tight-binding calculations.^24^ Making epitaxial connections might also lower the ionization threshold, enabling the free-electron state to be reached, and thus, metallic transport may emerge.^83^

In a more general situation, the localization length scales with carrier density. Fu et al. theoretically predicted that ligand-capped QD solids can also achieve the MIT.^84^ However, the carrier density required to achieve this transition is experimentally unrealistic, since MIT is essentially a competition among all the disorder and tunneling matrix parameters (this is when the distance between QDs has to be considered). Therefore, the expectation of achieving band-like transport in QD solids can only be realized from the epitaxial connection system.

Since we can now define the critical carrier density to achieve band-like transport (metallic behavior), the low-temperature mobility in this state can be estimated as follows:^85^

5 $$\mu =\frac{3^{5/3}}{2\pi^{2/3}}\frac{e}{\hbar }\frac{\rho^{2}}{g^{2/3}n^{1/3}d}$$

Taking as an example the above-mentioned PbSe (*g* = 4) with carrier density equal to the critical carrier density (*n* = *n*_c_ = 2.8 × 10^20^ cm^–3^), the expected mobility is 5.5 cm^2^ V^–1^ s^–1^. Similarly, for a nondegenerate system such as CdSe (*g* = 1) with the same size and connection parameter, the estimated mobility at the critical carrier density (*n*_c_ = 6.9 × 10^19^ cm^–3^) is 22 cm^2^ V^–1^ s^–1^. Surprisingly, these estimated mobility values are not that high compared to some recent reports, in which mobility values up to hundreds of cm^2^ V^–1^ s^–1^ were claimed (Table 1). In a metallic-like semiconductor with a high carrier density or high doping level, the low-temperature transport is influenced by scattering (impurity scattering or carrier–carrier scattering), which can lower the mobility values.^94^

In practice, achieving carrier densities on the order of 10^14^ cm^–2^ or (10^21^ cm^–3^) using conventional solid-state gating with SiO_2_ dielectrics is challenging due to the limited gate capacitance. Thus, alternative strategies, such as doping, are often employed to achieve higher carrier densities required to induce metallicity. A notable experimental demonstration aligned with the theoretical perspective on the criterion of MIT in connected QD assemblies has been reported in networks of touching ZnO nanocrystals (NCs).^95,96^ In this system, the degree of interparticle contact was precisely controlled during synthesis by using atomic layer deposition (ALD), while the carrier density was modulated via photodoping. As the carrier density increased from about 2.2 × 10^19^  to 6.9 × 10^19^ cm^–3^, the temperature dependence of the conductivity shifted from a positive gradient to a nearly temperature-independent behavior at an experimental critical carrier density of 3.2 × 10^19^ cm^–3^ (*n*ρ^3^ = 0.71). The conductance approached that of bulk density polycrystalline ZnO, with a measured conductance *G* ≈ 1000 μS at highest carrier density, 2 orders magnitude higher than the quantum conductance limit (*G*_m_ = 80 μS), as shown in Figure 5b.^95^

An advanced approach to modulating charge transport involves using ionic liquids (ILs) as dielectric materials, a technique that is widely used, particularly in low-dimensional systems. The formation of an electric double layer (EDL) at the semiconductor surface provides an exceptionally high capacitance, typically 1–10 μF cm^–2^, enabling carrier density accumulation of up to 10^15^ cm^–2^.^97^ This concept, commonly termed the electric-double-layer transistor (EDLT), has also been applied to CQD solids. Such high carrier accumulation facilitates the filling of trap states, thereby allowing access to more intrinsic transport properties.^34,72,98^ Moreover, when the accumulated carrier density is sufficiently high, a transition from insulating (semiconducting) to metallic behavior can occur. This insulator-to-metal transition (IMT) has been reported across a variety of low-dimensional materials, including two-dimensional systems,^97,99^ nanowires,^100^ and organic semiconductors.^101,102^

The possible IMT has also been observed in epitaxially connected PbS QD superlattices. As shown in Figure 5c, the temperature-dependent four-terminal (4T) sheet conductance (*G*_S_) of EDLT-gated PbS superlattices exhibits a clear transition. At low gate voltages (<2 V), the system demonstrated an insulating behavior. On the other hand, the conductance becomes more temperature-independent and approaches the quantum conductance *G*_m_ (shaded area in Figure 5c) at a higher gate potential (∼2.6 V).^17^ Within this regime, the accumulated carrier density was greater than 30 e^–^/dot. This number should be sufficient to reach the 1*d*_e_ state, which is aligned to the MIT criterion.^85^ Additionally, an electron mobility in the range of 8–13 cm^2^ V^–1^ s^–1^ was obtained, which is coincidently on the same order of magnitude as the theoretical mobility (∼12 cm^2^ V^–1^ s^–1^) based on the MIT criterion at the critical carrier density (*n*_c_ ∼ 9.8 × 10^19^ cm^–3^). Notably, for EDLT measurement (or FET in general), the charge carriers are accumulated at the QD–ionic liquid interface, by which the electrostatic doping effectively forms only at a monolayer of QD assembly, making a 2D-like system.

Despite the high capacitance of ionic liquid gating, some technical drawbacks may limit the gating performance in EDLTs, especially at low temperatures. The ionic conductivity of ions decreases with decreasing temperature, and ions become immobilized below their freezing points. Therefore, the tunability of the gate voltage to field-induced charge accumulation using the ionic liquid is limited by its glass transition temperature, which differs from one ionic liquid to another, typically around 180–220 K.^103^ Below this glass transition temperature, the ions freeze, retaining the formed electric double layer if previously gated and maintaining the charge accumulation. Continuous tuning of the charge-carrier density at low temperatures becomes nearly impossible. However, to measure charge-carrier transport at different carrier densities at low temperature, most specialists introduce and adjust the applied gate voltage by raising the sample temperature to room temperature or slightly above the freezing or glass transition points, then cooling it again with a new value of accumulated charge carriers.^17,99,100,102^ Although it has some technical drawbacks, frozen ionic liquid also enables achieving much higher carrier densities beyond the ionic liquid electrochemical window by immediately freezing the formed EDL after application of a higher gate voltage.^97^

To date, most discussions of possible band-like transport in an epitaxially connected QD superlattice have focused on the role of Anderson localization due to structural and energetic disorder. On the other hand, recent studies have considered more intrinsic features that influence carrier transport between coupled QDs, such as electron–phonon coupling.^104–106^ Hou et al. showed that the carrier transfer in a neck QD is strongly affected by the lattice symmetry, orientational (preference) attachment, and connection width (Figure 5d). Among those parameters, the connection width plays a crucial role in determining the non-adiabatic and adiabatic carrier transfer regimes (Figure 5e). The non-adiabatic carrier transfer was demonstrated in a narrow connection width, which corresponded to the weak electronic hybridization between donor and acceptor sites and fast nuclear vibration. In contrast, coherent adiabatic carrier dynamics is achieved with a large connection width, which enhances the electronic hybridization of donor–acceptor states and suppresses nuclear vibrations, facilitating faster carrier transfer.^104^ Taken together the MIT criterion and electron–phonon coupling, these theoretical insights converge on a common principle: that enlarging the connection width provides a viable pathway toward realizing metallic or band-like transport in QD arrays, particularly in epitaxially connected QD superlattices.^104^

### Technical Assessment of Transport Measurement

So far, we have discussed the criterion to achieve band-like (metallic) transport in QD solids. In this subsection, we will discuss technical issues related to how to distinguish the hopping and band (metallic) conduction. According to the MIT criterion, the conductance of QD solids has to surpass the quantum conductance *G*_m_. So far, the claim of possible band transport in QD solids is indicated by a mobility basis. Carrier mobilities approaching or even exceeding 1 cm^2^ V^–1^ s^–1^ have been interpreted as suggesting band-like transport.^20,32,107^ This mobility approximation is based on theoretical considerations from studies in organic semiconductors due to perceived similarities in the localization characeteristics.^108^ However, this thinking framework has been argued and clarified in a previous discussion, in which the lattice parameter of an organic semiconductor cannot be applied to hybrid organic–inorganic systems such as QD solids.^27^ In addition, considering the contradiction on the predicted mobility at the MIT state and the reported high mobility (Table 1), this leads to ambiguity in defining the transport regime of CQD solids. Hence, the low-temperature transport should be considered in terms of conductance for clarity, instead of mobility.

Additionally, the overestimation of the carrier mobility may arise from an underestimation of the charge-carrier density when unconventional gating techniques are employed. This discrepancy occurs from variations in the method used to determine capacitance or carrier density. The number of mobile carriers depends on either ion penetration into the channel (electrochemical gating) or the formation of an electrostatic double layer, both of which are strongly influenced by the applied gate voltage and often demonstrate nonlinear behavior. Several approaches have been used to estimate carrier density, including (1) simplifying the carrier density by assuming a constant capacitance (e.g., the average capacitance of the analogue electrolyte capacitor, measured without applied gate bias or at only one specific bias, etc.),^20,32^ (2) gate displacement current measurements of the transistors,^109^ and (3) gate-voltage-dependent and time-dependent capacitance measurements.^17^ This carrier density estimation will affect the calculation of mobility, so overestimation or underestimation of mobility might result.

Moreover, the hopping transport typically demonstrates a positive gradient of temperature-dependent mobility or conductance (d*G*/d*T* > 1), where the conductance decreases as the temperature decreases due to the thermally activated nature of charge transport. At higher temperatures, electrons have sufficient thermal energy to hop between localized sites, thereby increasing the conductance. In contrast, a negative temperature coefficient of conductance is often associated with band conduction, in which conductance increases as the temperature decreases. However, this interpretation is not always straightforward in QD solids, particularly because phonon-assisted mechanisms govern hopping transport in these systems, as demonstrated by HgTe QD solids.^88^ For comparison, a systematic transition from insulating to metallic behavior has been demonstrated in organic semiconductors, where the resistance (1/*G*) gradually decreases with increasing doping level. A sign change of d*R*/d*T* occurred as the resistance hit below the quantum resistance limit (1/*G*_m_).^102^ Similar efforts have also been performed for QD solids.^17,96^

Band conduction can be further confirmed by the behavior of charge-carrier transport under a magnetic field, such as through Hall effect measurements. However, some might not fully agree with the idea that measuring the effect in a system with a polaronic-assisted hopping mechanism (according to Marcus theory) could yield reliable Hall effect parameters.^27^ In band conduction, the mobile charge carriers experience a Lorentz force (*F*_L_), which can contribute to the formation of the Hall potential (*V*_Hall_). On the other hand, in pure thermally activated hopping transport, this Hall potential is absent. Hence, the question arises as to where the transition from insulator to metallic (or vice versa) occurs or whether the system exhibits hopping conduction with some fraction of charge-carrier delocalization or band-like transport.

The existence of the Hall effect in hopping conduction had been a subject of intense discussion,^101,110–112^ which was later applied to the percolation of charge carriers in organic (polymer) conductors.^113^ For instance, Yi et al. developed a model for Hall effect response in organic conductors, in which the existence of hopping carriers contributes to the underdeveloped Hall effect.^114^ Suppose that we have contributions of delocalized and hopping carriers. Then the total conductivity involves all mobile carriers:

6 $$\sigma =\sigma_{\mathrm{band}}+\sigma_{\mathrm{hopping}}=en_{\mathrm{band}}\mu_{\mathrm{band}}+en_{\mathrm{hopping}}\mu_{\mathrm{hopping}}$$

where μ_hopping_ is lower than μ_band_. Under the magnetic field, the mobile carriers from band-like conduction experience the Lorentz force (*F*_L_ = *e*μ_band_ *E*_*x*_ *B*, where *E*_*x*_ is the longitudinal electric field), creating a transverse Hall potential (*V*_Hall_). Meanwhile, the magnetic field does not work on the hopping carriers. As the Hall potential builds up, the formed transverse electric field (*E*_*y*_) interacts with the hopping carriers, causing it to migrate in the opposite direction to that of band-like conduction. This condition compensates for the measured Hall potential, which might be smaller than the actual Hall potential when only pure band-like transport is present. Therefore, the Hall effect measurement might yield an overestimated carrier density (or an underestimated carrier mobility) because hopping conduction is present in the system. However, if the system is evaluated through gate modulation in an FET, the contribution of hopping carriers can be determined by comparing the carrier density (or mobility) obtained from the Hall effect (*n*_Hall_, μ_Hall_) measurement with that from gating modulation (*n*_FET_, μ_FET_). Indeed, this can only be done qualitatively since the carrier density and mobility estimated from gating alone are assumed to be ideal (without trap states at the interface). According to the proposed model by Yi et al.,^114^ the measured *n*_Hall_ can be much greater than *n*_FET_ in a strong hopping regime, and thus, μ_Hall_ < μ_FET_. Meanwhile, *n*_Hall_ ≈ *n*_FET_ (or μ_Hall_ ≈ μ_FET_) is attributed to the intermediate hopping contribution and is usually associated with decent delocalized carrier transport.

Several works have reported Hall effect responses in CQD solids.^40,88,91,93^ This observation highlights the possible delocalization of the charge carriers. The first report was on all-inorganic InAs NC solids.^40^ The claimed Hall mobility was ∼16 cm^2^ V^–1^ s^–1^ with a negative gradient of temperature-dependent mobility around the room-temperature regime. Similarly, Hall effect response has been observed in Hg-based QD solids, with Hall mobility reaching ∼5 cm^2^ V^–1^ cm^–1^.^88^ Both systems demonstrated a monotonically decreasing mobility at lower temperatures, indicating polaron-assisted hopping transport behavior despite the clear observation of a Hall signal. According to the discussed model, this can happen as long as a delocalized carrier is present that contributes to the total conduction, even though the contribution of the hopping carrier might be more substantial, resulting in *n*_Hall_ greater than *n*_FET_ (or μ_Hall_ < μ_FET_).^114^ In addition, according to the MIT criterion, the band-like transport can be achieved when the 1p_e_ state is fully occupied (corresponding to approximately 40 e^–^/dot for Pb-based QDs and ∼10 e^–^/dot for Cd- or Hg-based QD). This implies that in the aforementioned examples of Hall effect measurements in QD solids with low doping levels, charge transport was likely dominated by the hopping process, and the measured Hall response may require careful interpretation. Similar observation of the Hall effect in a strong hopping regime has been demonstrated in an organic (polymer) conductor, where the Hall response was measurable even though the insulator-to-metallic transition has not been achieved.^101,112^

Alternatively, the behavior of charge carriers in a magnetic field can be probed through the longitudinal resistance, commonly called magnetoresistance. In systems undergoing a metal-to-insulator transition due to Anderson localization, magnetoresistance provides valuable insight into the level of disorder and whether the transport falls within the weak or strong localization regime.^115^ To build intuition, we first consider systems with band transport, such as metals or highly doped semiconductors with negligible impurities or defects. In these materials, charge carriers form an electron gas that exhibits ballistic transport. As impurities or defects are introduced, electron scattering becomes more frequent, and transport gradually crosses over to the diffusive regime.^116^ With increasing disorder, multiple scattering events lead to random quantum interference, which can exponentially localize the electron wavefunction and ultimately drive a metal-to-insulator transition.^117–119^ Importantly, this interference-induced localization can be partially suppressed by an external magnetic field, resulting in a reduction of resistance, known as negative magnetoresistance.

In contrast, charge transport in colloidal QD assemblies typically occurs in a highly disordered regime (strong localization). However, improved electronic coupling can gradually promote more delocalized charge carriers, as expected in epitaxially connected QD superlattices. If Anderson localization arising from structural disorder is indeed the dominant transport mechanism in this system and if the conductivity approaches or even exceeds the quantum conductance, one would expect the emergence of negative magnetoresistance. However, magnetoresistance measurements should only be interpreted once key conditions are satisfied, such as the formation of highly ordered superlattices and the attainment of the predicted critical carrier density.^85^ Otherwise, the observed magnetoresistance response may be strongly influenced by other intrinsic effects, including spin-related interactions or magnetic confinement, rather than by weak-localization-induced transport mechanisms.^120,121^

## Conclusions and Future Outlook

To summarize, enabling band-like transport in CQD solids is possible, though challenging, particularly due to the inevitable energetic disorder from a typically 5% to 10% size distribution.^10^ The metallic-to-insulating transition is a competition between the disorder parameters and tunneling matrix, including the coupling energy between adjacent QDs. In the weak coupling regime (i.e., when the separation between QDs is large), the coupling energy is much smaller than the disorder parameters, leading to very short localization length. To achieve MIT in this condition, a huge amount of charge density is required that experimentally is impossible to be reach.^84^ On the other hand, in the case of epitaxially connected QD superlattices, the coupling energy is enough to counter the disorder parameters, which can reduce the threshold of carrier density to achieve MIT.^17,85,96^ One may question whether structural disorder in epitaxially connected QD superlattices gives rise to Anderson localization in the same manner as in continuum band-transport materials with a high density of impurities or defects. Analogously to the bottom-up and top-down phenomena, crossing over at weak Anderson localization is an intermediate regime.

Furthermore, in evaluating carrier transport in QD solids, the mobility is not a robust indicator to justify the transport mechanism (hopping or band-like transport). So far, the benchmark for transport in CQD solids is an organic (polymer) conductor, which shares a common hopping behavior. However, there are still some differences between organic molecules and inorganic QDs. Hence, the standard of “minimum” mobility for prospecting hopping or band-like transport should be reconsidered. Instead, systematic and integrated low-temperature conductivity and Hall-effect measurements are necessary to clarify the transport mechanism.

Moreover, the discovery of colloidal QD superlattices offers an opportunity to perform band engineering, as they exhibit “giant atom” behavior, where the band structure may differ depending on how they are assembled.^24,25^ In most cases, the emerging quantum transport is demonstrated from a unique band structure, which also derives from an unconventional atomic lattice. For instance, some intriguing quantum transport phases were found in long-range-ordered moiré superlattices of twisted bilayer graphene or other two-dimensional materials.^122^ Indeed, it cannot be a direct and fair comparison to QD assemblies. However, the crucial idea here, which can be imitated, is to obtain long-range order with a highly coherent electron wavefunction within the entire superlattice and low disorder parameters. Hence, researching the realization of large-scale coherent QD superlattices is worthwhile.

In principle, achieving carrier mobilities exceeding 1 cm^2^ V^–1^ s^–1^ at room temperature is already sufficient to realize optoelectronic devices based on any short-ligand- or inorganic-capped CQD solids, even though charge-carrier transport in these systems remains governed by hopping mechanisms, underscoring the challenge of balancing their optical and electrical properties. However, a deeper question remains: what is the true driving force behind the ongoing pursuit of band-like transport in semiconductor CQDs? In other words, is band-like transport truly necessary? The answer will depend on perspective and motivation, particularly whether the goal is technological advancement or fundamental understanding. For instance, in photopumped and indirectly electrically pumped CQD-based lasers, charge transport is not a critical factor as long as carriers can be efficiently injected into the QD and recombine efficiently.^123^ In fact, individual CQDs are often kept as isolated as possible to suppress Auger recombination,^124^ and the same concept applies to CQD-based light-emitting devices.^3,46,123,125^

Realizing long-range superlattices might reduce disorder and enhance electronic coupling.^17,68,70^ This might cause the transfer integral to exceed the disorder parameters, including the charging energy.^82^ Hence, the threshold to reach a free-electron state of an excited electron theoretically becomes more achievable, particularly in the system with Auger ionization present, thus promoting large photoconductivity.^83^ It will be beneficial to realize more efficient photovoltaic devices and photodetectors.^126^ Furthermore, the enhanced carrier mobilities will significantly improve charge extraction, translating the high internal quantum efficiency of CQD solids into a usable power conversion efficiency. This realization can be achieved by transforming current assembly methods to enable the formation of a reliable 3D stack of epitaxially connected QD superlattices with adequate thickness.

Significant progress in CQD solids has already enabled the development of optoelectronic devices that combine strong optical responses with reasonably good charge transport. From an application-driven perspective, however, the pursuit of band-like transport may hold particular value in realizing highly coherent, epitaxially connected QD superlattices. Notably, a new direction for exploiting CQD solids emerged less than a decade ago, shifting the emphasis from optical properties to charge transport. It led to the proposal of QD-based thermoelectric devices,^127,128^ which convert heat into electricity via the Seebeck effect under a thermal gradient. The efficiency of thermoelectric devices is typically quantified by the dimensionless figure of merit *ZT* = *S* ^2^σ*T*/κ, in which *S*, *T*, σ, and κ are the Seebeck coefficient, absolute temperature, electrical conductivity, and thermal conductivity, respectively. A high *ZT* requires simultaneously maximizing *S* and σ while minimizing κ. However, these parameters are inherently coupled, making the optimization of *ZT* highly challenging. Hence, strategies to decouple electrical and thermal transport are crucial. One promising approach is nanoscaling, which (i) enhances the Seebeck coefficient by tailoring band structures and density of states (DOS)^129,130^ and (ii) suppresses thermal conductivity through increased phonon scattering at particle or domain boundaries^131^ if (iii) sufficiently high electrical conductivity can be maintained. These advantages are naturally embodied in CQD solids. Despite their potential, reports on CQD-based thermoelectrics remain limited.^132–136^ The primary challenge lies in achieving adequate electrical conductivity, as conventional ligand-capped CQDs often impose severe transport bottlenecks. Thus, epitaxially connected QD superlattices hold promise for enhancing thermoelectrics. In addition, the presence of Anderson localization within such superlattices may facilitate the decoupling of electrical and thermal conductivity, thereby implicitly suggesting a potential improvement in the thermoelectric figure of merit *ZT*.^137^

On the other hand, the intensive investigation of epitaxially connected QD superlattice systems is still limited to rock-salt (cubic) crystal structures, as demonstrated by Pb–chalcogenide-based CQDs because of their well-defined faceting as truncated cuboctahedra. The other “model compounds” in the colloidal QD field, Cd–chalcogenides and Hg–chalcogenides, have truncated tetrahedral structures. Lower point-group symmetry leads to greater entropy loss upon ordering, making it more challenging to form long-range-ordered superlattices. Nevertheless, efforts to form epitaxially connected QD molecules have been performed to find routes for larger assemblies.^138–140^ Furthermore, numerous new CQDs with more diverse chemical compositions and crystal structures have been developed,^141–144^ including core–shell nanocrystals.^107,125,136,145^ Controlling such diverse surface conditions is challenging, and new strategies to enable well-defined assemblies must be considered. Moreover, combining two or more types of QDs might offer new approaches to designing superlattices with an emerging band structure.^58,146–148^ Last but not least, advanced theoretical, calculation, modeling, and AI/ML supports are expected to provide new insights into possible emerging transport in CQD building blocks with different assembly structures.

## ‡

D.S.: Currently on leave from the Department of Metallurgical Engineering, Institut Teknologi Bandung, Indonesia.
